# Risk determinants in early intervention use during the first postnatal year in children born very preterm

**DOI:** 10.1186/1471-2431-13-201

**Published:** 2013-12-05

**Authors:** Margo A Pritchard, Paul B Colditz, David Cartwright, Peter H Gray, David Tudehope, Elaine Beller

**Affiliations:** 1 Women’s and Newborn Services, Royal Brisbane Women’s Hospital, Herston 4029, Australia; 2 The University of Queensland Centre for Clinical Research, Herston 4029, Australia; 3 Mater Research Institute-University of Queensland, Mater Health Services, South Brisbane 4101, Australia; 4 Bond University, University Drive, Robina 4229, Australia; 5 Department of Paediatrics and Child Health, The University of Queensland, Brisbane, Australia

**Keywords:** Neurodevelopment, Preterm infant, Early intervention

## Abstract

**Background:**

Early interventions (EI) are recognised for their potential risk-reduction capacity. Although developmental delay is common in children born very preterm reports continue to suggest poor uptake of EI services. This study examined the risk determinants of EI in Australian children born less than 32 weeks gestation during the first year of life.

**Methods:**

As part of a multi-centre-randomised-trial, 195 children were prospectively studied during their first year of life and EI use, type of follow-up, perinatal, social and parental psychosocial risk factors were collected using questionnaires. Child neurodevelopmental disability-status was assessed at 12-months (cerebral palsy, blind, deaf, developmental quotient 1standard deviation (SD) below mean). The associations between EI and variables were examined using Pearson’s chi-squared test (χ^2^) and regression techniques.

**Results:**

A total of 55% of children received EI, 51% attended post discharge neonatal intensive care unit (NICU) and the remainder attended exclusive primary health care. Risk factors included, 50% perinatal, 19% social and 34% psychosocial and at 12-months 23% were categorised as disabled. Low social risk and NICU follow-up attendance were significantly associated with EI use but only perinatal risk (OR 3.1, 95% CI 1.7, 5.6, *p* = <0.01) and disability (OR 2.2, 95% CI 1.1, 4.7, *p* = 0.04) independently predicted EI use.

**Conclusions:**

It is reassuring that children with perinatal risk receive EI, opportunity remains to improve EI uptake in families with social and parental psychosocial risk during the first year of life.

## Background

Recent studies demonstrate an increase in the prevalence of very preterm birth (VP = <32 weeks gestation) and an accompanying extensive range of developmentally based lifespan disabilities [1-3]. Preventing premature birth and its consequent disabilities remains elusive with child health surveillance systems an important health strategy to identify and intervene in children at risk for adverse development. Whilst our understanding of biologic and social experience in early risk mechanisms for poor development in the preterm population is incomplete, [4-7] there is growing evidence early intervention (EI) can mediate risk and improve lifespan outcomes [8-10].

Accordingly, recent Organisation for Economic Co-operation and Development [11] and World Health Organization [12] initiatives provide state-based universal access to early childhood development programs through an increasingly broader range of preventative individual and community interventions with targeted and treatment level components. The focus on early risk-reduction [13-15] means that EI services are more than conventional delay/disability needs-based intervention and now emphasize risk-prevention efforts. Readily available in most communities is access to quality professionally-delivered broad-based family support, standardised parent education, training, counselling, mental health management and infant physical therapies. Although evidence-based, eligibility criteria and focus on treatment or prevention varies, it is likely that that their uptake will be significant in the preterm population.

Recent guidelines and quality-of-care indicators for developmental follow-up of preterm children now reflect an awareness of early medical, social and psychosocial risk identification and interventions during the first year of life [16,17]. Of 70 indicators, endorsed by the American Academy of Pediatrics (AAP), 83% are primary care and 70% are applicable to children during their first year of life of which 14% are for psychosocial assessment. There is emerging evidence that the outcomes and health service engagement in children born preterm is mismatched with many children failing to receive both preventative and treatment services in early infancy. To date, little is known about the risk factors associated with EI use during the preterm child’s first year of life when health care and surveillance is often shared between tertiary neonatal unit follow-up and primary health care.

The purpose of this study is to evaluate the use of EI in children born less than 32 weeks gestation during their first post discharge year of life. We examined the relationship of EI use with i) common surveillance risk factors (perinatal, socioeconomic and maternal psychosocial), and type of post discharge health care and ii) the child’s disability status at 12 months (ca) for prematurity. An increased understanding of very EI use may provide insights on early surveillance practices in preterm children.

## Methods

This present study is part of a Queensland multi-site (Royal Brisbane Women’s Hospital, Mater Mothers’ Hospitals and The Townsville Hospital, Australia) randomised study to assess the efficacy of primary and tertiary health care assessment in identifying developmental status in 202 children born VP at 12-months and has been previously reported [18]. Human Research Ethics Committees approved the study protocol at each hospital and at The University of Queensland Medical Research Ethics Committee (Project Number 2002000895). Written informed consent was obtained from parents. Data were available for 195 of the 202 (97%) children longitudinally studied from birth to 12-months corrected age (ca). We collected information on a range of risk factors known to be associated with child development and which can be routinely screened for in both primary and tertiary health care. We included information on perinatal and socioeconomic risk from discharge case notes and maternal psychosocial risk by validated questionnaire at 6 weeks post partum. During the study period, parents recorded if they attended neonatal unit follow-up or primary health care and any EI they attended. The child’s disability status was determine at 12-months (ca) using standardised medical and psychometric assessment as previously reported [18].

### Outcome measure

The categorical outcome was EI and defined as use of any individualised or centre based parenting, physiotherapy, physical, occupational, behavioural, family, nutritional and developmental education or therapy. We included all intervention types that were used by the family and did not differentiate between treatment or preventive intervention.

### Predictor variables of the early intervention use

We developed three risk categories drawn from the AAP quality indicators for neurodevelopmental follow-up of very low birth weight children. Perinatal risk was defined as having any intraventricular haemorrhage, periventricular leukomalacia, chronic lung disease, failed physiological hearing status, retinopathy of prematurity and any ongoing metabolic or surgical issue. Socioeconomic risk included a family having any of the following factors; single parent family, maternal education at junior-high level, the lowest quintile of gross household income, or indigenous. Psychosocial risk included either an Edinburgh Postnatal Depression Scale (EDPS) [19] screening score >12 indicating depression and a Parental Stress Index–Short Form (PSI-SF) [20] total stress score ≥85 indicating parenting stress. Post-discharge health care was defined as either receiving exclusive primary health care or tertiary neonatal unit outpatient care with or without primary health care.

### Child disability status

A composite outcome to determine a disability status included a neurological examination and motor assessment for cerebral palsy, developmental impairment < -2SD below the mean on a standardised psychometric test assessed with the Revised Griffith Mental Development Scales [21] or the Bayley Scales of Infant Development II [22] or deaf requiring hearing aids. A sub classification of disability was used to distinguish the mildly disabled (mild developmental impairment with a developmental score between < -1 SD and -2 SD) children from the moderate-severe disabled (developmental impairment with a developmental score < - 2SD) children and has previously been described [18].

### Analysis

The Chi-Square Test (χ^2^) compared the categorical predictors and child’s disability status against the categorical outcome (Received EI vs. No EI). Logistic regression analysis examined the association between EI and risk variables, adjusted for by baseline characteristics (gestational age < 28 weeks, multiple birth and male gender). Data were dichotomised with results reported as odds ratio (OR) with 95% confidence intervals (CI) and *p*-values. The statistical software used was SPSS for Windows (version 20.0, SPSS Inc., Chicago, IL, USA).

## Results

The baseline characteristics of the infants were within the reported range for children of less than 32 weeks gestation. There were more multiple births and children born with a gestational age less than 28 weeks in the EI use group. There were no gender differences between the EI groups (Table 1). Overall 55% (108/195) of children received an EI. Forty nine percent (96/195) of children attended exclusive primary health care and the remainder attended neonatal unit outpatient care either exclusively or with some primary health care. The 12-month disability rate was 23% (45/195) including 20 moderate-severe and 25 mild cases. Rates for socioeconomic risk (19%, 37/195), perinatal risk (50%, 97/195) and psychosocial risk (34%, 66/195) were present. There were 134 (69%) children with at least one risk factor of which seven (5%) had all three risks and 54 (40%) had two risk factors.

**Table 1:** Baseline characteristics of children by early intervention group

**Variable**	**Received EI**	**No EI**	**OR**	**95% CI**	***p *** **value**
	**N = 108 (%)**	**N = 87 (%)**			**N = 195**
Gestational age <28 weeks	49 (45.4)	22 (25.3)	1.6	1.2, 2.4	<0.01
Multiple birth	45 (43.5)	25 (28.7)	1.5	1.1, 2.1	0.03
Male gender	53 (49.0)	47 (54.0)	1.2	0.7, 2.1	0.49

Children who had received EI had higher rates of perinatal risk, lower socioeconomic risk and were more likely to have attended post discharge neonatal follow-up rather than primary care (Table 2). All 20 children with moderate-severe disability received EI during the 12 months with 60% (12/20) having received neonatal follow-up rather than exclusive primary care and 90% (18/20) having a perinatal risk. Fewer children with mild disability received EI or had a perinatal risk 52% (13/25) although a similar proportion received neonatal follow-up 48% (12/25).

**Table 2:** Risk factors, surveillance type and disability status for children receiving early intervention services

**Variable**	**Received EI**	**No EI**	**OR**	**95% CI**	***p *** **value**
	**N = 108 (%)**	**N = 87 (%)**			**N = 195**
Perinatal risk	68 (63.0)	29 (33.3)	1.8	1.4, 2.8	<0.01
Socioeconomic risk	15 (13.9)	22 (25.3)	0.7	0.5, 0.9	0.04
Psychosocial risk	37 (34.3)	29 (33.3)	1.1	0.7, 1.4	0.80
Post discharge surveillance-NICU	62 (57.4)	37 (42.5)	1.8	1.1, 3.2	0.03
Disability status- none	75 (69.4)	74 (85.0)	0.8	0.6, 0.9	<0.01
All disabled	33 (30.6)	12 (13.8)	1.9	1.1, 3.1	<0.01
Mild disabled	13 (12.0)	12 (13.8)	1.0	0.7, 1.6	0.87
Moderate-severe disabled	20 (18.5)	0 (0)	-	-	

Logistic regression confirmed, that even after adjusting for the type of post-discharge surveillance and baseline characteristics, perinatal risk was the only independent risk predictor of EI through the first year of life (OR 3.1, 95% CI 1.7, 5.6, *p* = <0.001). In addition, compared to children without a disability, those with a disability at 12-months were more likely to have received EI (OR 2.2, 95%CI 1.1, 4.7, *p* = 0.04).

## Discussion

In our sample of children born VP, perinatal risk alone was associated with receiving EI during the first year of life independently of whether children attended neonatal clinics or primary care health services. It is conventional, in many countries, to enrol preterm and other high-risk categories of children into follow-up developmental programs for formal diagnostic assessment for early childhood disability. Prior to that time children with high-risk characteristics, most often medical risk, are seen in neonatal clinics whilst the remainder attend primary care facilities. In our study, it was not surprising to see children with perinatal risk were more likely to attend neonatal clinics during their first year of life. Similar to other studies [23], we found that the sensitivity of perinatal risk was high, for children with a disability at 12-months (70%) although the specificity was low (52%). It is likely that both clinicians and parents had better understanding of the benefits of EI based on perinatal, rather than social or psychosocial, risk as an accepted pathway to EI.

Neither of the environmental risks predicted EI use during the first year of life. Like other studies, our univariate analysis showed an inverse relationship between socioeconomic risk and receiving EI services [24]. This is a complex finding and may be related to the reduced competency and health literacy experienced in resource-restricted families. It is well recognised that this group are difficult to follow-up and their children experience poor development [25,26]. Conversely, there is some evidence that parents perceive clinicians as having difficulty assessing and addressing social and psychosocial problems [27].

Similar to other neonatal units, Queensland units provide primary care clinicians a comprehensive summary, often with a care plan, primarily emphasising disease-based morbidity that is likely to facilitate risk identification and the need for EI use. Over one third of Queensland children are discharged from neonatal units to regional or remote districts where primary health often has well developed community based programs aimed to support the family and child development. Additionally, whilst the stability and prediction of disability remains poor during early childhood, developmental malleability of perinatal and environmental risk factors through EI is potentially high. Reliance on effective primary health care as a pathway to EI is particularly important where potential barriers associated with geographical regionalisation and resource restrictions are identified.

### Limitations of the study

The strength of this current study is its use of a contemporary cohort and the investigation of the use of a broad definition of EI and its use in the immediate after hospital discharge care period. Generalization of our results in the context of health surveillance in developing nations may also be useful at the conceptual level. Screening studies in developing countries have postulated a causal relation between environmental factors and child outcomes highlighting the need for screening beyond medical and physical function [28]. One main limitation is our aggregation of EI services which precluded analysis of preventative versus treatment use as well as an understanding of both service quality and evidence-based versus ad hoc and used due to availability. In addition, disability rates beyond infancy may also offer further insight into EI uses. Despite the limitations, this study demonstrates which risk factors are related to EI use and which risk factors may be overlooked during the early infancy period.

## Conclusions

Identifying and intervening modifiable risk in very early child development through routine NICU and primary care surveillance practice requires consideration of the effectiveness of early surveillance. This geographical based study has shown that children born VP attend both primary and tertiary health care services prior to their routine early childhood developmental assessments. However, children with social and psychosocial risks are much less likely to receive EI compared to those with perinatal risk. Pediatric health workers are uniquely placed to provide early and ongoing screening and identification of a broad range of risks essential for appropriate EI and ensuring the effectiveness of early surveillance practice in high-risk populations. Increasing both the tertiary and primary sector knowledge and practice of well validated developmental, social and psychosocial screens and techniques may help improve the identification of high risk families and children and a greater likelihood for the referral to preventative and treatment EI.

## Competing interests

The authors declare that we have no competing personal or financial interests.

## Authors’ contributions

EB participated in the design of the study and performed the statistical analysis. MAP conceived of the study, and with PBC, PHG, DT contributed to its design and coordination of study sites and participants. All authors read and approved the final manuscript.

## Pre-publication history

The pre-publication history for this paper can be accessed here:

http://www.biomedcentral.com/1471-2431/13/201/prepub
